# Neonatal End-of-Life Symptom Management

**DOI:** 10.3389/fped.2020.574121

**Published:** 2020-09-11

**Authors:** DonnaMaria E. Cortezzo, Mark Meyer

**Affiliations:** ^1^Division of Neonatal and Pulmonary Biology, Cincinnati Children's Hospital Medical Center, Cincinnati, OH, United States; ^2^Division of Pain and Palliative Medicine, Cincinnati Children's Hospital Medical Center, Cincinnati, OH, United States; ^3^Department of Pediatrics, University of Cincinnati College of Medicine, Cincinnati, OH, United States; ^4^Department of Anesthesiology, University of Cincinnati College of Medicine, Cincinnati, OH, United States; ^5^Division of Anesthesia, Cincinnati Children's Hospital Medical Center, Cincinnati, OH, United States

**Keywords:** Perinatal palliative care, neonatal, end-of-life, symptom management, pharmacological management

## Abstract

**Purpose of review:** Despite advances in technology and treatment options, over 15,000 neonates die each year in the United States. The majority of the deaths, with some estimates as high as 80%, are the result of a planned redirection of care or comfort measures only approach to care. When curative or life-prolonging interventions are not available or have been exhausted, parents focus on preserving quality of life and eliminating needless suffering. Parents hope their child will have a peaceful death and will not feel pain. A significant component of end-of-life care is high quality symptom evaluation and management. It is important that neonatal providers are knowledgeable in symptom management to address common sources of suffering and distress for babies and their families at the end-of-life (EOL).

**Recent findings:** Medically complex neonates with life-threatening conditions are a unique patient population and there is little research on end-of-life symptom assessment and management. While there are tools available to assess symptoms for adolescents and adults, there is not a recognized set of tools for the neonatal population. Nonetheless, it is widely accepted that neonates experience significant symptoms at end-of-life. Most commonly acknowledged manifestations are pain, dyspnea, agitation, and secretions. In the absence of data and established guidelines, there is variability in their clinical management. This contributes to provider discomfort and inadequate symptom control.

**Summary:** End-of-life symptom assessment and management is an important component of neonatal end-of-life care. While there remains a paucity of studies and data, it is prudent that providers adequately manage symptoms. Likewise, it is important that providers are educated so that they can effectively guide families through the dying process by discussing disease progression, physical changes, and providing empathetic support. In this review, the authors make recommendations for non-pharmacological and pharmacological management of end-of-life symptoms in neonates.

## Introduction

Neonatal end-of-life care is pivotal for the patient, parents, and providers. Whether the need for end-of-life care is the result of a comfort measures only approach with a known life-limiting diagnosis, the result of redirection of care after a trial of therapy, or the end result despite maximal invasive medical interventions, it is an incredibly difficult time filled with emotions and uncertainty. At a time typically filled with joy and hopes for the future they will have with their child, parents of neonates at EOL are grieving the loss of their baby even before death occurs (1). Parents note that when goals have transitioned to end-of-life care, they worry that their child will experience pain and physical suffering. They hope for a peaceful death with minimal discomfort for their child (2–8). One of the focuses of palliative care is the prevention and alleviation of symptoms including physical, emotional, and perceived suffering of the patient (9). In addition to providing support, it is paramount that providers focus on pain and symptom management (10, 11). At the same time, there can be many unknowns. For clinicians as well as families this is a time of uncertainty. It is often difficult to predict the timing of death, whether it be hours, minutes, days, or more (8). Effective management requires anticipation of the disease manifestations as well as the severity of symptoms for each patient. This can vary greatly and is often related in part to the underlying diagnoses and conditions leading to death. For any patient population, anticipating the symptom burden can be challenging even with a strong understanding of the pathophysiology. There is an added layer of complexity when caring for neonates as they cannot communicate their symptoms or express how they are feeling. For older children, adolescents, and adults, the gold standard of symptom assessment is self-report. However, there is no such standard that exists for preverbal patients. In neonates, there are few reliable tools to accurately assess symptoms and observational measures can be blunted depending on the gestational age and underlying disease process. This makes it difficult for providers to know if a neonate is experiencing symptoms at the EOL and if interventions are alleviating the symptoms to a satisfactory and acceptable level. In spite of all of these challenges, it is pertinent that providers are able to assess and treat symptoms a neonate may have at EOL so that the baby, the parents, and family have a peaceful experience.

## Scope of Neonatal End-of-Life Care and Symptom Management

In the United States, there are over 15,000 neonatal deaths each year (12). The majority of these deaths are the result of congenital malformations or chromosomal anomalies, with up to 3% of pregnancies being complicated by a fetal life-limiting diagnosis (13–15). While some families opt for a comfort measures approach to care at home, the majority of neonatal deaths occur in the neonatal intensive care unit (NICU). Regardless of the location of care, most deaths, with some estimates as high as 80%, are the result of a comfort measures only approach to care or a redirection of care after a trial of therapy (16). As a result, there is a unique opportunity to thoughtfully and methodically plan to treat symptoms based on the anticipated needs unique to the pathophysiology effecting each neonate (8, 17–20).

Likely in part due to the paucity of data, there is significant variability in neonatal EOL comfort practices. A recent survey of members of the American Academy of Pediatrics Section on Neonatal-Perinatal Medicine inquired about neonatal end-of-life practices across America (21). Of the respondents, nearly half did not have institutional guidelines for comfort care. Of those that did, 80.9% addressed pain management and over 75% discussed non-pharmacological management. Other symptoms, such as secretions and anxiety, were addressed less than half the time.

## End-of Life Symptoms

Symptoms experienced during EOL care that can be applicable to the neonatal population include agitation, dyspnea, neuroirritability, pain, and increased secretions (20, 22). Agitation and neuroirritability, generally speaking, are unpleasant states of arousal. They can be the result of the underlying disease process or from interventions and medications. Common manifestations include autonomic signs, increased motor activity, restlessness, and disturbed or disrupted sleep. Dyspnea is discomfort with breathing and is largely a subjective experience or sensation. Though subjective, a majority of verbal patients report dyspnea at the end-of-life and family members frequently perceive the patient is having dyspnea (23–26). As a result, it is reasonable to associate physical signs of increased work of breathing and tachypnea with the subjective experience of dyspnea at EOL. Pain, defined as an unpleasant sensation that results in discomfort and distress, is commonly experienced by pediatric patients in the last days of life (19, 22, 23, 26). The etiology and degree of pain experienced can vary greatly. It is important to know that there are different types of pain. Nociceptive pain is the result of tissue damage or inflammation and can be somatic, localized to a specific region, or visceral, affecting the internal organs. Neuropathic pain is the result of damage or irritation to the nerves. Identifying the type of pain that is occurring and understanding the etiology and underlying mechanism can help direct more effective approaches to therapy. Much of the early work of Anand was focused on understanding the neuroanatomy, neurophysiology, and neurochemistry of pain in fetuses and neonates (27). At 20–22 weeks gestation fetuses have developed their peripheral pain sensors and the neuronal migration of the ascending pathway to connect with the thalamus and sensory cortex has occurred. The peripheral nervous system and spinal cord are well-developed by 30 weeks gestation. The descending pathways develops later. This would indicate that neonates as young as 22 weeks gestation have the same number of pain receptors as an adult on a much smaller surface area and without the complete development of the inhibitory pathways. In addition, substance P and other neurotransmitters can also cause excitation of uninvolved neurons leading to lower pain thresholds and more robust, increased and longer pain responses in even the youngest neonates. These findings were accompanied by recognition in the clinical setting of pain responses and led to the acceptance the neonates are capable of experiencing pain and a change in practice around assessing and treating pain in neonates. With the understanding and acceptance that neonates experience pain, it is appropriate to assume that neonates should be properly monitored for signs of pain and treated accordingly (27–30). Excessive secretions are not as commonly seen but can be experienced if the neonate is unable to effectively swallow. This can be the result of the underlying disease process or from overall decreased tone, alertness, and activity during the dying process.

## Assessment of Symptoms

While there are approved and validated methods to evaluate EOL symptoms for adults and older children, there are limited data and methods for neonates (20, 22, 31). Considering the clinical circumstances surrounding death, neonates may not be able to mount the normal physiologic response to pain that is seen in older populations. Similarly, neonates may not display other symptoms in the same manner other individuals do, making it difficult for providers to assess and effectively manage them. As a result, the methods and tools utilized in adolescents and adults cannot be translated to this population. The evaluation is largely reliant on clinician and family perception of symptoms. A recent qualitative exploratory study interviewed NICU nurses about the EOL care their patients received (32). While small, this study displayed themes of uncertainty, discomfort, and chaos. Nurses were not always certain, based on the physical signs they were seeing and on the clinical situation, if their patient was experiencing pain or any other symptoms. Lack of education, resources, and consistency in the approach to EOL care contributed to overall provider discomfort. In addition, providers may be focused on other tasks such as supporting the family which lend to subtle physical signs going unnoticed (8).

These challenges, along with historically limited documentation, makes it difficult to draw conclusions or to come to a consensus about standardized approaches to symptom assessment in neonates. One study of 20 neonates with planned redirections of care described the documentation of symptoms exhibited and the treatment of those symptoms during the dying process (8). The authors noted that all patient documentation had missing data related to EOL care and that there was a varying amount of detail with regard to the infant's symptoms and use of medications. Specifically, no pain scores were documented after invasive medical interventions were withdrawn or care was redirected. With gaps in documentation, providers may not be able to accurately assess the baby. Furthermore, this leads to significant limitations in research. Without detailed documentation regarding symptoms and interventions, there are challenges in drawing conclusions about current assessment and management of neonatal EOL care, as well as challenges in developing innovative or consistent guidelines and approaches to that care.

Pain, one of the most common symptoms discussed at EOL in older populations, has been historically challenging to assess in neonates. Additionally, there is concern that pain has been underappreciated and undertreated at baseline in this population. Research shows that fetuses and neonates have well-developed peripheral nervous systems and pain-related neurotransmitters are present as early as 22 weeks gestation; it has been accepted that neonates, even when born premature, can experience pain (27, 33, 34). In recent years, there have been improvements in assessment and management of neonatal pain, especially post-operatively (28, 29, 34–37). While not specific to EOL care, it is pertinent that providers utilize a neonatal specific pain assessment tool (9, 20). Carter describes at length the various pain assessment tools available for neonates and concludes that the evidence does not allow for recommendation of one specific tool but that using a validated tool is pertinent (20). It is pertinent that providers have been trained to use the tool and are experienced with it to increase the reliability of the reported results. An ideal tool is one that is validated in both premature and term neonates for subacute and chronic pain. Given the challenges of assessing pain in neonates, strong tools combine both behavioral and physiological data in the assessment. Common pain assessment tools suitable for this population are COMFORTneo, CRIES (Cry, Requirement for more oxygen, Increased vital signs, Expressions, Sleeplessness), N-PASS (Neonatal Pain, Agitation, and Sedation Scale), NIPS (Neonatal Infant Pain Scale), and PIPP (Premature Infant Pain Profile) (20, 38). While there is a paucity of data regarding pain and comfort in neonates at the end-of-life, one study showed that significantly fewer neonates had their pain evaluated with a pain assessment scale routinely used in other pediatrics patients receiving end-of-life care (20, 39, 40).

Other symptoms, such as agitation, dyspnea, and secretions are evaluated based on the clinical circumstance and physical assessment. Agitation or neuroirritability are suspected when there is self-reported or perceived increased motor activity, restlessness, and disturbed sleep. Similarly, assessment of dyspnea is reliant on a subjective report either by the patient or a visual assessment by another individual based on markers such as respiratory rate, the presence of hypoxia, and work of breathing.

To date, there remains little research on symptom assessment, symptom management, and the evaluation of interventions used in EOL care of neonates (41). Although there is a lack of standardized assessment tools or standards for interventions for neonatal EOL care, some providers advocate for treating common distressing conditions at the EOL in neonates whether or not they appear to be in distress. In these conditions, older, verbal populations commonly experience a high symptom burden from the same sources of suffering and their self-report and observations scores correspond to the severity of their burden. Providers suggest that neonates experience the same symptom burden and ought to be treat similarly (17, 42). For example, in verbal pediatric patients there is a high likelihood of dyspnea occurring after ventilator withdrawal, and there is also a high likelihood of dyspnea causing additional secondary symptoms that can be alleviated with pain medications (43). The same can be assumed with neonatal populations experiencing similar distressing symptoms after going through similar clinical experiences.

### Parent Perception

Parental perception of suffering is extremely important as parents know their baby better than anyone and are sensitive to signs of pain or distress others may not be aware of. Parents may perceive their baby is distressed by physical changes they observe during the dying process. They often equate these changes with pain or discomfort (32, 44, 45). As symptom assessment has a component of subjectivity, there can be differing opinions from those interacting with the baby. In fact, one small study of EOL experiences for neonates revealed that 85% of providers felt symptoms were well-managed while only 57% of parents felt their baby was comfortable (44). Another study asked the bereaved parents of 40 neonates about their observations of symptoms during the last week of life (46). Mothers and fathers were given a list of 22 symptoms and on average the mothers reported 6.63 symptoms in their baby's last week of life while the fathers reported and average of 5.67 symptoms. Mothers most often noted respiratory distress, agitation, and pain while fathers most often noted respiratory distress, agitation, and lethargy. Both reported respiratory distress as the worst symptom in severity and the most difficult symptom to watch as they perceived it as the most uncomfortable symptom. On average parents reported their baby experienced a moderate amount of suffering. Only 6 of the symptoms inquired about where routinely documented in the medical record and of those there was no significant difference between parenteral or provider report. While parents and providers may not agree on the extent of suffering, provider initiated discussion around EOL care with parents allows for improved care of the neonate and reduction in anxiety of parents and possible tension between parents and the care team.

## Management of End-of-Life Symptoms

While there is a body of evidence and guidance for the treatment of EOL symptoms in adults and adolescents, there is limited data for neonates (20). As a result, there is a great deal of variability in pharmacological and non-pharmacological end-of-life symptom management for neonates (32, 47, 48). While there is variability, it is important that symptoms including pain are addressed by both pharmacological and non-pharmacological means (49). Other symptoms seen that may need to be addressed include dyspnea, agitation, neuroirritability, and secretions (20, 22).

### Non-pharmacological Management

Preparing the family for what to expect during the dying process helps to alleviate parental anxiety. As a result, effective communication with families that supports the parental role and conveys empathy is required. Providers must prepare families for the dying process with clear and honest conversations, realizing that families will want varying degrees of information about the physiologic changes (9, 49). By understanding the changes that may occur, some families are less distressed by symptoms such as gasping and color changes (50, 51). They are able to appreciate that the physical changes occurring do not necessarily mean their child is experiencing dyspnea. They feel more prepared for the death, have less grief, and are more satisfied with the care their child received (2, 52–54). One study of 131 bereaved caregivers of children who died of cancer revealed that they did not feel prepared for the medical needs of their child as they approached death (55). Parent who felt unprepared for the changes at the time of death perceived their child suffered more. They expressed that they desire clear, honest communication about the medical issues around the time of death. Without an understanding of these changes, they may feel powerless or unsure of what their child is experiencing. By fostering open communication, providers can set expectations, address misconceptions, and encourage the family to convey when they are concerned that their baby is having symptoms so that they are addressed in a timely fashion. This is important not only for the comfort of the baby and the parent's experience with their child, but also for how the parents process the death of their baby. Parent perception of suffering has a lasting impact that can prolong and complicate their grief. Parents who are uncertain about what to expect at the EOL have more severe grief afterwards (56, 57).

Care should be taken to reduce uncomfortable procedures or invasive touching/stimulation (58). When symptoms occur, interventions such as decreasing stimulation, massage, skin care, mouth care, elevating the head or repositioning for dyspnea, fluid restriction, and gentle suctioning may be beneficial (9, 42, 49). Gentle suction and repositioning, for example, are more effective than pharmacological therapy the majority of the time for secretion management and noisy breathing. Swaddling, skin-to-skin contact, and non-nutritive sucking can be beneficial for agitation and pain. These measures help to meet the basic needs of neonates and promote positive bonding experiences (59). Families appreciate the opportunity to bond with and parent their baby (60). Eighteen nurses in Scandinavia were interviewed about their practices around skin-to-skin contact for parents and neonates during EOL care (61). Skin-to-skin interaction is known to decrease pain, improve labored breathing, and have positive psychosocial effects. Providers believe facilitating this care promotes comfort and bonding for both the mother and baby. This, in conjunction with promoting emotional and physical intimacy for families, can be very effective in alleviating dyspnea and agitation as hypoxia and hypercapnia that can occur during this time also have natural sedative effects (39). While there is expressed knowledge of the benefit of non-pharmacological interventions, documentation of their use in the medical chart remains limited (8). This makes it difficult to know the true extent to which these techniques are utilized and are effective.

### Pharmacological Management

Non-pharmacological interventions will often alleviate some but not all symptoms neonates experience at the EOL. If anticipated and discussed ahead of time, parental apprehension and resistance to using medications to treat symptoms can be alleviated. Historically, providers have been hesitant to administer medications, specifically opioids and benzodiazepines, out of concern that they will hasten death. This concern of providing medications that have respiratory depression as a side effect in a medically fragile neonate has led to inadequate treatment of symptoms (39). However, there is a commonly accepted ethical notion known as the principle of double-effect which states that an action is justifiable if the nature of the act is good, the good effects are the intended effects as opposed to the bad effects, and that the good effects outweigh the bad given the circumstances (20, 47). Along these lines, it is considered good medical practice and standard of care to alleviate symptoms and pain during the dying process. In addition, multiple studies have shown that administering opioids and sedatives in appropriate doses to treat EOL symptoms does not hasten death, carries little risk of respiratory depression, and increases perceived comfort during the dying process (20, 62, 63).

While there remains variability in practice around administering medications for EOL symptoms in neonates, many providers give some type of medication. Typical classes of medications utilized are opioids, benzodiazepines, antipyretics, anticholinergics, diuretics, hypnotics, and anticonvulsants (9, 42, 64). The majority of studies in neonates are limited to intravenous administration in a hospital setting. A study of 20 neonates with planned redirections of care noted that most babies received pharmacological therapy. Eighty-five percent received opioids or benzodiazepines prior to withdrawal of invasive medical interventions and 60% received them afterwards. The majority received intravenous medications with one receiving oral medications, and five receiving aerosolized forms (8). One of the earliest studies looking at practices around neonatal EOL care reviewed deaths over a 3 year period in the NICU secondary to withdrawal of or withholding invasive medical interventions (18). Eight-four percent of neonates were given opioid analgesia. When care was redirected in the setting of perceived suffering or with major surgical or congenital anomalies, infants were more likely to receive opioids, most commonly morphine or fentanyl. If deaths outside of the delivery room were excluded, birth weight had no impact on the likelihood of receiving analgesia. Doses of opioids given were consistent with typical the pharmacological range when taking in to account previous opioid exposure. There was no significant difference in the time from withdrawal of invasive medical interventions to death for those who received no opioids or <0.2 mg/kg of morphine compared to those who received >0.2 mg/kg of morphine. In the institution at that time, the nurses would routinely ask physicians for opioid analgesia if they felt the neonate was in pain based on their assessment. The use of benzodiazepines was not typical and as a result there was no use of them documented in the study.

Other studies similarly found that there was no difference in birth weight or gestational age in whether or not infants received medications for symptoms as part of EOL care and that the vast majority of patients who died in the delivery room did not receive opioids (39). A study of 171 neonates at a level III NICU showed that 27.5% did not receive opioids or benzodiazepines for EOL care (17). Those in the group weighing <800 g were less likely to receive these medications. However, those weighing >1,500 g did not receive pain medications as often as those weighing 800–1,500 g. It was thought that potentially those in the 800–1,500 g group more often had diagnoses associated with pain. The fact that any died without comfort medications, though, was concerning to the authors.

One study retrospectively reviewed practices around medication administration for neonates at four NICUs in the United States, Canada, and the Netherlands (47). Of note, similar to previous studies, none of the babies who died in the delivery room received medications. Outside of the delivery room setting, most neonates received some type of medication for comfort, an opioid and/or benzodiazepine, in the 48 hours prior to redirection of care and after the redirection of care. Benzodiazepines were utilized more frequently in the NICUs in the United States than in the other countries. However, only the NICUs outside of the United States increased the dose of comfort medications prior to redirection of care. If a neuromuscular blockade was already being used, it was discontinued at two of the centers but continued at the other two. Only the NICU in the Netherlands administered a neuromuscular blockade after redirection of care and that was only to relieve suffering at the request of family.

While it is well-known that there is significant variability in the assessment and approach to treatment of pain in neonates in other countries, there is little evidence with regards to variations in practices around EOL care for neonates. The largest difference in the literature around EOL practices in other countries is related to euthanasia. While a rare occurrence, there are instances where the Dutch and Belgium consider euthanasia of neonates to be acceptable. The prognosis must be certain, there must be evidence of undue suffering, and parents must give consent. While this topic is sensitive, complex, and wrought with ethical debate, it is an acceptable practice for the Dutch and Belgium under rare circumstances that are tightly regulated (65–67).

### Classes of Commonly Used Medications

Alpha 2 adrenergic agonists, clonidine, and dexmedetomidine, have analgesic and sedative properties. They been used in the neonatal population, particular after cardiac surgery, for some time (36). These options are appealing as they are usually well-tolerated and have minimal effect on respiratory drive.

Anticholinergics block acetylcholine either centrally or peripherally. As a result, they can cause fever, tachycardia, and other physiologic symptoms. In proper doses these side effects can be minimized and the effect of anhidrosis can be beneficial. Specific to EOL care this can reduce the amount of secretions. It is important to note, though, that there is little evidence that pharmacological therapy is effective for secretion management or the death rattle during the dying process (20).

Anticonvulsants have properties that lend themselves to EOL care in certain circumstances. Seizures themselves are unlikely during the dying process unless there is a preexisting condition. However, some of these medications can be effective at treating agitation or neuroirritability. Gabapentin, which works on the voltage dependent calcium channel, can be beneficial for neonates with difficult to control agitation or neuroirritability (68–71).

Benzodiazepines work by activating the gamma aminobutyric acid A receptors. They are commonly used in neonates for sedative purposes and have no analgesic properties. Side effects can be myoclonic jerks, hypotension, and, at higher doses, respiratory depression, or excessive sedation. In end-of-life care, they can be used to treat agitation and dyspnea.

Cyclooxygenase-2 enzyme inhibitors, acetaminophen and non-steroidal anti-inflammatory drugs, have been well-studied in the neonatal population and as a result have been used to treat fevers and mild pain, or as an adjuvant for pain treatment plans. They could have similar roles in end-of-life symptom management. Non-steroidal anti-inflammatory medications convert arachidonic acid to prostaglandin and are known to have anti-inflammatory, analgesic, and antipyretic properties. They can inhibit platelet function and lead to renal impairment. If already part of an analgesic treatment plan, they would likely be beneficial to continue for EOL care.

Dissociative anesthetics such as ketamine, have amnetic, analgesic, and sedative properties. Ketamineis is not known to cause hypotension or respiratory depression in neonates. However, there have been concerns at higher doses in animal models of apoptosis which has led to limited use until recently in the neonatal population (72, 73). In human models and in the presence of painful stimuli, it has been shown to have a possible protective effect (74). Given the limited data, its use had been limited to invasive procedures. In opioid exposed patients, it could be beneficial to treat pain, dyspnea, and agitation at the EOL.

Opioids are a class of medications that act on the opioid receptors in the body. Depending on the specific medication, they work on the mu, kappa, and delta portion of the receptors with varying degrees of affinity. They have effective analgesic and sedative properties and are readily used in neonates (29, 36). They are most commonly used for and most effective in treating pain, dyspnea, and agitation during the dying process.

### Routes of Administration

Historically, in the hospital setting, IV access has been maintained though EOL care as a stable way to provide comfort medications either as continuous or intermittent infusions (64). Both methods can lead to adequate symptom control but some providers feel intermittent infusions, if possible, may make it easier for families to hold their babies and may be more conducive to memory-making. As for subcutaneous drug administration in neonates, there is very little data (75). Recently, there has been a focus on alternative and less invasive routes to provide medications for EOL care. The reasoning is largely to remove invasive equipment such an intravenous needles, aid in memory-making, allow EOL care to occur outside to the NICU, and to allow the option for EOL care in the home setting while still effectively managing symptoms. The route of administration with the most data and experience in these circumstance is oral administration. There are well-established dosage guidelines of oral medications in neonates. Alternately, while oral transmucosal (buccal or sublingual) administration provides several benefits, there are very few medications concentrated or designed for this route of administration making its utility limited (76). One center utilized the limited data available to develop a protocol for neonates receiving EOL care (76). Medications used included morphine, midazolam, lorazepam, scopolamine, and glycopyrrolate with doses equivalent to intravenous dosing. There were clear criteria on when to administer the medications and which dosages to use. While compliance with utilizing the pain assessment tool was low, by report, all survey respondents felt symptoms were controlled with oral transmucosal administration and no adverse side effects were noted. The vast majority said they would recommend this protocol. Similarly, a small retrospective study looking for non-invasive methods of controlling symptoms examined intranasal fentanyl in neonates (77). This medication and mode was evaluated because of the rapid onset of action, ease of administration, and because opioids are utilized to treat two of the most common symptoms-pain and dyspnea. There is more data available on the intranasal route of administration in children as opposed to the sublingual or buccal route. Doses were similar to intravenous doses while accounting for incomplete bioavailability. In the 11 neonates who received intranasal fentanyl for treatment of EOL pain or dyspnea, they received an average of 4.5 doses and had symptom control without the need for intravenous medications. There were no noted side effects and some were able to be cared for in settings outside of the NICU. Another route that is at times utilized in the neonatal population is transdermal. While there are transdermal forms of opioids and alpha 2 agonists available, there are is limited data of their use in neonates to make clear recommendations. The dosages in which these medications are available may be one limitation to the use in the neonatal population.

## Palliative Sedation

While it is accepted and expected that symptoms should be appropriately managed during EOL care, there is a clear distinction between appropriately managing symptoms and providing the medication with the intent of ending life or hastening death (78–80). This is particularly true when there is a high symptom burden refractory to typical medications and doses. One article looking at Dutch practices found that the intent is not always obvious by reviewing the type and dose of medications used (78). In this nationwide study, the authors utilized retrospective chart review and physician interviews to describe the type, dose, and reason for medication administration surrounding EOL care in the Dutch NICUs. Comparable to other studies, roughly 14% did not receive medications. They found that doses of opioids and sedatives were generally increased following EOL discussions and that documentation of the reasons for this were present <50% of the time despite the physicians explaining the reasoning in interviews. Per interviews, medications were usually titrated to mitigate or prevent symptoms such as pain, agitation, and gasping. In 10% of cases the intent was to hasten the dying process. Opioids were administered above what they considered normal dosages in 5% of patients before EOL decisions were made and in 17% afterwards. Benzodiazepines dosages were above the considered normal dosage in 12% of patients after EOL decisions were made. Roughly 16% of neonates received neuromuscular blockades as part of the EOL care. In interviews reasons included to prevent gasping, per parental request, and to limit suffering. While there has been debate over the years around the use of neuromuscular blockade at the EOL, it is generally accepted that they should not be utilized after redirection of care as the principle of double effect typically cannot be justified and it would bring the patient to his death (9, 81). In cases where a paralytic has recently been given, if time is not allowed for the paralytic to wear off, there should be discussions with the family, the intent should be to relieve suffering, and it should be clear that the burden of waiting an extended period of time for the effects of the paralytic to completely wear off would significantly outweigh the benefits (9, 20, 47). Another study from Europe examining the use of medications with the intent of hastening death in neonates concluded that the medications used are highly effective, the infants were moribund at the time they received the medications, and there was a discrepancy in documentation of the reported intent and effect (79). Furthermore, they felt that it is not easy to distinguish between intent to end life and provide adequate symptom control during the dying process.

When sedation is utilized at the EOL as a means to decrease and control symptom burden, it has been referred to as terminal sedation, palliative sedation therapy, controlled sedation for intractable patients, EOL sedation, and continuous sedation for the dying (82, 83). Palliative sedation can occur across a continuum from anxiolysis to deep sedation with loss of consciousness. As pediatric palliative care has evolved, the field is able to articulate the terminology, objectives and methods to provide effective and ethical management of severe symptoms at the EOL. Providing an effective level of symptom control to match the symptom burden without hastening death through respiratory depression is the objective. The provision of palliative sedation has been supported by numerous professional organizations including the American Medical Association, National Hospice and Palliative Care Organization, American Academy of Hospice and Palliative Medicine, and American Academy of Pediatrics.

Many pediatric palliative care providers apply criteria for patients under consideration for palliative sedation prior to initiation of sedation including limited prognosis of hours to weeks, confirmation that the target symptom is refractory, parental consent, and a goal of comfort care and foregoing resuscitative efforts. Accurately determining prognosis can be challenging for providers and discussion with specialists and colleagues can produce an accurate perspective. While not often utilized in the NICU, palliative sedation may have a role for patients who have refractory symptoms and/or a lengthy history of exposure to pharmacological therapies typically used to treat EOL symptoms. The latter often results in tolerance and resistance to conventional symptom management. Beyond light sedation, palliative sedation can be classified as proportionate palliative sedation and palliative sedation to unconsciousness.

### Proportionate Palliative Sedation

Proportionate Palliative Sedation (PPS) is symptom management that directly targets a specific symptom with a medication's identified mechanism of action while accepting that the side effects of the medication will cause sedation. Pain treated with analgesia is a common scenario in which the symptom is managed with a class of medications that directly targets the symptom. Opioids, for example, provide analgesia and can induce sedation at higher doses. Opioid analgesics directly reduce the suffering caused by pain while the use of hypnotics, such as midazolam, would mask the manifestations of the pain. Alternatively, seizure management, a less common threat to comfort at the EOL, can be complicated by antiepileptic therapy that can induce sedation at levels needed to optimally treat refractory seizures. In PPS the antiepileptic therapy that targets the source of suffering is appropriate and the end-point is relief of suffering while causing the minimum amount of sedation necessary.

### The Level of Sedation and Titration

Sedation may vary from intermittent to continuous as the patient may waken spontaneously or may not waken once sedation is initiated. The depth of sedation can vary from light in which the patient readily wakens to voice and light touch to deep. In the latter, the patient is unable to be awake at all. In deep sedation, respiratory depression may be marked by decreased respiratory rate, tidal volumes, and obstructive breathing. In all scenarios, the objective is to provide relief of symptoms. The process for achieving adequate dosing is based upon medication titration proportional to the severity of the symptoms. When a symptom breaks through a previously achieved level of control, dosing increases can be based on the severity. Many providers use the following guide to make medication increases that are proportional to the severity: mild breakthrough −10 to 20% increase, moderate breakthrough −20 to 30% increase, and severe −30 to 50% increase. Any increase in dosing, whether intermittent dosing or continuous infusion, is followed by a period of reassessment to ensure the symptom has been addressed. The term Palliative Sedation Therapy (PST) has been advocated for to describe the active management that is required to adequately treat evolving symptoms in that frequent assessment of symptoms coupled with titration of medications to achieve the balance is needed. This active management process involves parents and family by including them in the symptom assessment.

### Palliative Sedation to Unconsciousness (PSU)

There are clinical circumstances in which proportional palliative sedation is inadequate and suffering persists despite adequate titration and dosing. If the symptom is refractory, obtaining comfort can be achieved by using agents that alter the level of consciousness but do not directly reduce the symptom itself. PSU is provided using classes of sedative and hypnotic medications in conjunction with the medication targeted to treat the specific symptom. Distinct from PPS, the intent is to induce loss of consciousness to eliminate suffering. Agents commonly used included propofol, phenobarbital, ketamine, and dexmedetomidine. The addition of a hypnotic is often done only after all measures to treat symptoms have been exhausted and is administered in an addition to the current regimen.

## Discussion

Appropriate EOL symptom management, while challenging at times, is important for neonates. Every individual deserves to have their symptoms addressed so they can have as peaceful of a dying process as possible. Not only does it improve comfort for the neonate, it also improves the EOL experience for the family. This allows them to focus on spending time with their baby and make meaningful memories which can help lead to less complicated grief and a positive experience for the limited time they have with their baby. While there are challenges associated with identifying and quantifying some of the symptoms that neonates experience during the dying process, many providers and families report common symptoms including respiratory distress, pain, and agitation. These are the same EOL symptoms that are often experienced in adolescents and adults.

It is important, especially when there is time such as with a planned redirection of care, that the healthcare team clearly communicates with the family about the dying process. Clear communication can lead to an open dialogue to address some of the concerns and fears of the family. By understanding the changes they will see in their baby, they can better prepare themselves and begin to understand the difference between physiologic changes and signs of distress or discomfort. When they perceive less suffering surrounding the death of their child, they have less guilt and less complicated grief.

**Figure 1:**
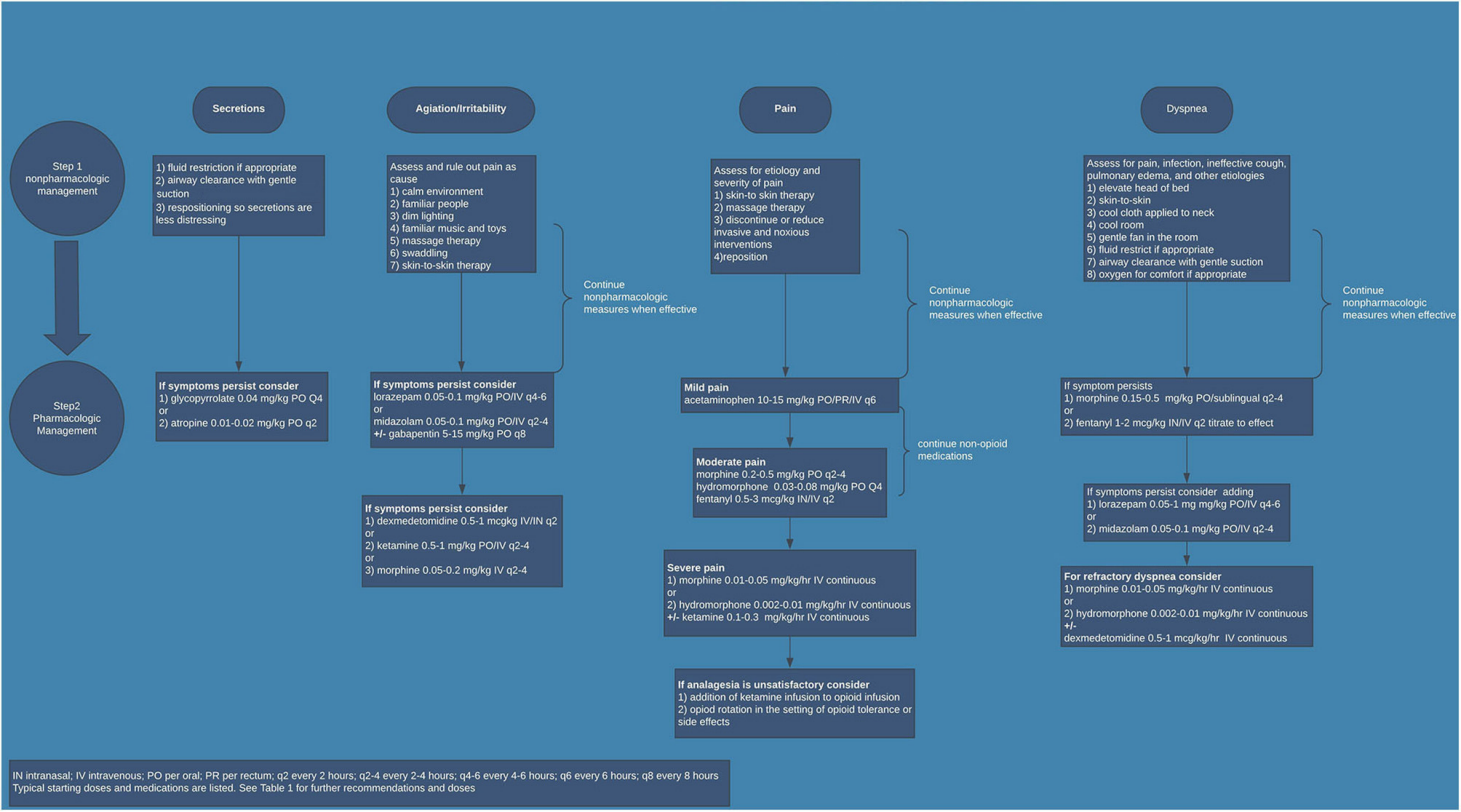
Neonatal end-of-life symptom management.

It is pertinent that symptoms be regularly assessed during the dying process. If EOL care takes place in the NICU, routine assessment by staff should occur. If EOL care occurs outside of the NICU, family and staff should be educated as to the common signs of agitation, dyspnea, neuroirritability, pain, and increased secretions. For agitation and neuroirritability, signs of restlessness, abnormal movements, and disturbed sleep should be monitored closely. With dyspnea, common associations in a non-verbal patient are tachypnea and increased work of breathing. For secretions, increased saliva coming out of the mouth, gagging, and the “death-rattle” are common signs. Pain is the symptom that has the most validated assessment tools in the neonatal population. While not specific to EOL, institutions should have an agreed upon tool routinely used for preterm and term neonates that evaluates chronic and subacute pain. If EOL care is occurring outside of the hospital, family should be educated about ways in which their baby manifests signs of pain or discomfort. Despite the challenges associated with adequately assessing symptoms in neonates, when symptoms appear they should be adequately treated in a multidisciplinary approach. Many of the therapies that are beneficial for treating these symptoms in adults have been utilized in neonates with perceived benefit. Environmental and non-pharmacological factors are incredibly important. Promoting bonding, holding, and skin-to-skin contact is therapeutic for both the neonate and family. Implementing interventions such as decreasing painful procedures, decreasing stimulation, ensuring normothermia, and repositioning can be quite effective. Often times, though, non-pharmacological interventions are not adequate. This is particularly true outside of the delivery room or in situations where a neonate is being compassionately extubated. Identifying if there is a treatable cause of the symptom is important in developing an effective treatment plan. For example, dyspnea that is secondary to a pneumonia may be treated differently than dyspnea secondary to congestive heart failure. Based on current literature, institutional expertise, and clinical experience of the authors, suggestions for common medications are included (see Table 1). Figure 1 is an algorithm for a practical application of and approach to EOL symptom management in the NICU population. Medication choices and doses used may need to be adjusted based on prior exposures. For example, if the neonate is already on an opioid drip the dose may need to be increased. At times, higher doses are needed to achieve adequate symptom management or alternative medications need to be utilized. In rare circumstances, where there is a high symptom burden and traditional treatment options have not achieved appropriate symptom control, palliative sedation may be appropriate. In either of these situations, if the provider is not comfortable or familiar with alternatives such as ketamine, dexmedetomidine, or gabapentin, or if the provider is not experienced with providing palliative sedation, expert input should be sought ahead of time.

**Table 1:** Neonatal end-of-life symptom management: suggested pharmacologic medications.

**Medication**	**Class**	**Symptom**	**Starting dose (per kg) with route and frequency**	**Comments**
Acetaminophen	COX2 inhibitor	Fever Mild pain	15 mg PO/PR q6 6–8 mg IV q8	As an adjuvant for pain
Atropine	Anticholinergic	Secretions	0.01–0.02 mg PO q2	No strong evidence
Dexmedetomidine	Selective alpha 2 agonist	Agitation Pain	0.5–1 mcg IV/IN q2 0.5–1 mcg/kg/hr IV continuous	
Fentanyl	Opioid	Pain Dyspnea	0.5–2 mcg IN/IV q2 1–4 mcg/kg/hr IV continuous	Quicker onset of action
Gabapentin	anticonvulsant	Agitation Neuroirritability	5–15 mg PO q8	
Glycopyrrolate	Anticholinergic	Secretions	0.01–0.02 mg IV q4 0.04–0.1 mg PO q4	No strong evidence
Ketamine	Dissociative anesthetic	Agitation Pain	0.5–1 mg PO/IV q2-4	
Lorazepam	Benzodiazepine	Agitation Dyspnea	0.05–0.1 mg PO/IV q4-6	
Methadone	Opioid	Pain	0.05–0.2 mg IV/PO q12-24 (initially q4 for 3 doses)	
Midazolam	Benzodiazepine	Agitation Dyspnea	0.05–0.1 mg IV q2-4 0.2–0.3 mg Sublingual q2-4 0.25 mg IN q2-3 0.05 mg/kg/hr IV continuous	Short acting
Morphine	Opioid	Pain Dyspnea Agitation	0.05–0.2 mg IV/IM q2-4 0.15–0.5 mg PO/Sublingual q2-4 0.01–0.05 mg/kg/hr IV continuous	

*COX-2 inhibitor, Cyclooxygenase-2 enzyme inhibitor; IN, intranasal; IM, intramuscular; IV, intravenous; PO, per oral; PR, per rectum; q2, every 2 h; q2-4, every 2–4 h; q4-6, every 4–6 h; q6, every 6 h; q8, every q hours; q12-24, every 12–24 h*.

*It may not be appropriate to continue feedings. They can be difficult to digest and cause fluid overload*.

Despite the recent focus on neonatal palliative care, there remains a scarcity of data in the literature about EOL symptom assessment and management in neonates. Some providers are still concerned about the possibility of medications causing respiratory depression and hastening the dying process. Studies have not shown this to be the case and it is accepted that it is ethically appropriate and medically necessary to treat EOL symptoms. While this is true, there are disagreements about the classes of or doses of medications to use and providers are left with little guidance on pharmacological management. This contributes largely to the wide variations in practices. Future research is needed to develop standardized tools to assess EOL symptoms in neonates. This would help providers and families have an objective way to evaluate symptoms. Beyond that, further research on the pharmacokinetics and pharmacodynamics and efficacy of the medications used is needed to better guide dosing recommendations and treatment options for these patients. A better understanding could lead to better symptom control and alternative medications, as well as alternative routes for administration. This could potentially lead to more choices for the location of EOL care for certain neonates.

With further research, institutions could develop more comprehensive guidelines for the assessment and management of EOL symptoms in neonates. In order to accomplish this, clinicians must take care in documenting their assessment of symptoms surrounding EOL as well as the interventions used. This includes noting the indication for the intervention and how effective it was at alleviating the symptom. This will lead to overall better control of symptoms, more consistency in the care provided, and less discomfort among staff and parents. One approach would be to develop an EOL symptom flowsheet in the electronic medical record where the various symptoms are listed along with the established mode of assessment. In this flowsheet, staff can easily document if symptoms are present, their severity, interventions used and a reassessment of the symptoms after interventions. This will provide valuable insight for patient care and for research. Even now in the absence of clear data for assessment and management of EOL symptoms in neonates, institutions should have guidelines in place. As part of that process, there should be education for staff around the dying process, EOL symptoms, and symptom management. With a better understanding, provider distress can be decreased. It also serves as an avenue to increase comfort and empower providers to have discussions with families around EOL care. While these discussions can be difficult, they promote a unified understanding of what to expect as well as shared decision-making that can ultimately translate to a better EOL experience for the neonate and the family.

## Author Contributions

DC conceptualized and designed the report, drafted the initial manuscript, and approved the final manuscript as submitted. MM aided in designing the manuscript, contributed to the content, and approved the final manuscript as submitted. All authors contributed to the article and approved the submitted version.

## Conflict of Interest

The authors declare that the research was conducted in the absence of any commercial or financial relationships that could be construed as a potential conflict of interest.

## Abbreviations

NICU: Neonatal Intensive Care Unit
EOL: end-of-life
PPS: Proportionate Palliative Sedation
PST: Sedation Therapy
PSU: Palliative Sedation to Unconsciousness.
